# Mapping of Diabetes Susceptibility Loci in a Domestic Cat Breed with an Unusually High Incidence of Diabetes Mellitus

**DOI:** 10.3390/genes11111369

**Published:** 2020-11-19

**Authors:** Lois Balmer, Caroline Ann O’Leary, Marilyn Menotti-Raymond, Victor David, Stephen O’Brien, Belinda Penglis, Sher Hendrickson, Mia Reeves-Johnson, Susan Gottlieb, Linda Fleeman, Dianne Vankan, Jacquie Rand, Grant Morahan

**Affiliations:** 1 Centre for Diabetes Research, Harry Perkins Institute for Medical Research, University of Western Australia, Nedlands 6009, Australia; l.balmer@ecu.edu.au; 2 School of Medical and Health Sciences, Edith Cowan University, Joondalup, Perth 6027, Australia; 3 School of Veterinary Science, the University of Queensland, Gottan 4343, Australia; c.oleary@uq.edu.au (C.A.O.); m.reevejohnson@uq.edu.au (M.R.-J.); susanalisongottlieb@gmail.com (S.G.); d.vankan@uq.edu.au (D.V.); j.rand@uq.edu.au (J.R.); 4 Laboratory of Genomic Diversity, Center for Cancer Research (FNLCR), Frederick, MD 21702, USA; Marilyn.Menotti@gmail.com; 5 Laboratory of Basic Research, Center for Cancer Research (FNLCR), National Cancer Institute, Frederick, MD 21702, USA; davidvic@mail.nih.gov; 6 Laboratory of Genomics Diversity, Center for Computer Technologies, ITMO University, 197101 St. Petersburg, Russia; lgdchief@gmail.com; 7 Guy Harvey Oceanographic Center, Halmos College of Arts and Sciences, Nova Southeastern University, Ft Lauderdale, FL 33004, USA; 8 *IDEXX* Laboratories, East Brisbane 4169, Australia; belinda.penglis@bigpond.com; 9 Department of Biology, Shepherd University, Shepherdstown, WV 25443, USA; shendric@shepherd.edu; 10 Animal Diabetes Australia, Melbourne 3155, Australia; l.fleeman@AnimalDiabetesAustralia.com.au; 11 American College of Veterinary Internal Medicine, University of Zurich, 8006 Zurich, Switzerland

**Keywords:** diabetes mellitus, Burmese cats, susceptibility, single-nucleotide polymorphism, genetic markers, LIPH

## Abstract

Genetic variants that are associated with susceptibility to type 2 diabetes (T2D) are important for identification of individuals at risk and can provide insights into the molecular basis of disease. Analysis of T2D in domestic animals provides both the opportunity to improve veterinary management and breeding programs as well as to identify novel T2D risk genes. Australian-bred Burmese (ABB) cats have a 4-fold increased incidence of type 2 diabetes (T2D) compared to Burmese cats bred in the United States. This is likely attributable to a genetic founder effect. We investigated this by performing a genome-wide association scan on ABB cats. Four SNPs were associated with the ABB T2D phenotype with *p* values <0.005. All exons and splice junctions of candidate genes near significant single-nucleotide polymorphisms (SNPs) were sequenced, including the genes *DGKG, IFG2BP2, SLC8A1, E2F6, ETV5, TRA2B* and *LIPH*. Six candidate polymorphisms were followed up in a larger cohort of ABB cats with or without T2D and also in Burmese cats bred in America, which exhibit low T2D incidence. The original SNPs were confirmed in this cohort as associated with the T2D phenotype, although no novel coding SNPs in any of the seven candidate genes showed association with T2D. The identification of genetic markers associated with T2D susceptibility in ABB cats will enable preventative health strategies and guide breeding programs to reduce the prevalence of T2D in these cats.

## 1. Introduction

Type 2 diabetes mellitus (T2D) is a polygenic disease with complex inheritance [1,2]. Studies of T2D in humans have identified over 80 genetic variants associated with disease susceptibility, but most of these contribute a small proportion of overall risk of disease, and the detailed molecular basis of how these contribute to disease is not well understood [3]. Animal models can provide additional evidence of the role of genes in T2D. For example, a polymorphism in the melanocortin 4 receptor gene (MC4R) was associated with T2D in overweight domestic cats, similar to humans [4]. Animal models can also provide a valuable opportunity to identify novel T2D genes. We demonstrated previously that Australian-bred Burmese (ABB) cats are at increased risk of developing T2D, and exhibit features typical of human T2D [5,6], including inadequate insulin secretion (dysfunctional β cells); impaired insulin action (insulin resistance) [7]; late age of onset; risk factors such as obesity and physical inactivity; islet vacuolation and amyloid deposition. Clinical features of diabetes in ABB cats are similar to atypical T2D in humans, which is most common in African-Americans, and includes presentation with very high blood glucose concentrations [8,9]. Diabetic ABB cats respond to insulin in a very similar way to human patients. Affected humans present with a history of polyuria, polydipsia and weight loss, but otherwise have a T2D phenotype and profile, and frequently have a family history of T2D. These patients respond to initial insulin treatment within days to weeks with long-lasting, insulin-free, near-normoglycaemic remission [9]. Similarly, cats with diabetes have high remission rates (67 to >80%) resulting in normoglycaemia without the need for insulin, if tight glycaemic control is instituted soon after diagnosis [10,11].

In a study of a cohort of 12,576 cats, the prevalence of diabetes was significantly higher in ABB cats than in domestic short or longhaired cats (*p* < 0.001), with the incidence rising to 10% in Burmese cats over eight years old [5]. Other Australian domestic cat breeds exhibit a diabetes incidence of ~0.25% to 1% [12]. American, European-and ABB cats have had distinct breeding histories since the 1970s [13]. Burmese cats bred in New Zealand and the UK, from where the ABB cats are believed to have originated, also demonstrate increased risk of diabetes [14,15]. However, Burmese cats in other countries such as the US do not show such increased T2D incidence [16,17]. US-bred Burmese cats did have different incidence rates for other genetic diseases [18].

Type 2 diabetes in ABB cats likely arises from a genetic founder effect. Several population bottlenecks have been experienced in cat evolution, including domestication and subsequently in the creation of breeds (mostly within the last 50–150 years) [19]. Of most relevance is the recent bottleneck associated with the establishment of the breed in Australia in 1957 from a very small number of individuals, possibly as few as five [13,20]. Founder effects can result in otherwise rare variants being established in a population and have had great utility in discovery of disease genes in domestic animal species. Thus, the genetic architecture of the ABB cat population provides an opportunity to identify novel genetic variants mediating T2D susceptibility. Identification of such genes could increase our understanding of molecular events leading to T2D and would be immediately applicable in the veterinary setting by identifying cats at risk of diabetes and improving breeding programs. Preventative management could also be implemented for at-risk individuals via dietary changes, weight loss and therapies to control glycaemia.

We hypothesize that genetic variant(s) predisposing to T2D demonstrate a higher frequency in ABB cats compared to American Burmese cats, thus providing a unique opportunity for discovery of diabetes-associated genes. We performed an exploratory case-control genome-wide association scan (GWAS), followed by validation of candidate SNPs in an expanded cohort. A similar approach identified candidate genes in other diseases [21,22].

## 2. Materials and Methods

This study was conducted in accordance with the Declaration of Helsinki and the protocol was approved by the Ethics Committee of The University of Queensland (AEC Approval Number: SVS/040/10/NCI/ABBOTT, date: 150910 to 150912; AEC Approval Number: SVS/200/12/NESTLE PURINA/ABBOT ANIMAL HEALTH, date: 180712 to 180715; AEC Approval Number: SVS/256/13/MEDINCELL/NCI, date: 311013 to 311016).

### 2.1. Characterisation of Australian-Bred Burmese Cats

Two ABB cat cohorts were studied. The first (“exploratory”) cohort contained 10 cats diagnosed with T2D and 10 control non-diabetics with normal glucose tolerance. Of the diabetic cats, four were male. Samples were obtained for five of these from cat-specific practices in Brisbane, Australia, one individual from a general practice, and 4 individuals from a veterinary commercial diagnostic laboratory (*IDEXX*, Brisbane, Australia) that had received blood samples from veterinarians as part of the diagnostic work-up for cats that had clinical signs suggestive of diabetes.

Cats were classed as T2D if they were first diagnosed at 8 years of age or older based on hyperglycaemia (blood glucose concentrations over 15 mmol/L) and clinical signs of polydipsia and polyuria. At the time of sampling, 4 cats were in remission and maintained euglycaemia without insulin. Selecting cats above 8 years of age reduced the risk of including cats with type 1 diabetes, which develops at an earlier age [23] and is relatively rare [24]. Inclusion of cats with other types of diabetes causing insulin resistance, most commonly acromegaly, was unlikely as cats were treated with typical doses of insulin and were not reported to have consistent clinical and/or pathology findings of acromegaly [25]. Further, 5/7 cats had feline pancreatic lipase immunoreactivity (fPLI) below 8 μg/L [26] and two had values below 12 μg/L and therefore not consistent with a diagnosis of active pancreatitis [27]; 7/8 tested had normal total thyroxine (TT4) and one had hyperthyroidism (TT4 107), one had liver disease, 3 had mildly increased liver enzymes, and 4 had renal dysfunction.

The 10 control ABB cats (five males) were all older than 8 years and exhibited fasting and 2 h blood glucose concentrations in a glucose tolerance test within reference ranges for age-matched cats. They were clinically healthy based on history, physical examination, routine haematological, routine biochemical, feline pancreatic lipase, and TT4 testing. For the glucose tolerance test, cats were fasted overnight and 0.5 g/kg 50% glucose injection BP was injected via a cephalic catheter placed 3 h before and samples collected from the pinna or paw at 0, 2, 3 and 4 h. All cats had a 2 h glucose concentration <10 mmol/L (upper 95% for normal reference range for glucose concentration 2 h following 0.5 g/kg glucose) and all blood glucose concentrations had returned to <6.5 mmol/L (upper limit 95% reference range for fasting concentration) by 2 to 2.5 h. All control cats had fPLI measurements <7.6 μg/L, 8 of 8 tested had normal TT4, and all had normal haematology and biochemistry.

The second cohort consisted of 84 ABB; 37 with T2D and 47 controls. Of the T2D cases, 24 were male and 13 female. Eight were from a diabetes-specific practice in Melbourne and 4 from a cat-specific practice in Brisbane. Of these latter 12 cases, 5 were in remission and the remaining 7 were stable on insulin doses <1.5 U/kg. Evidence of renal dysfunction was present in 5/7, hyperthyroidism in 1/5, and liver disease in 1/7. Five of these 12 cats had fPLI values available, with 4 being below 8 μg/L. The remaining 25 samples were obtained Australia-wide by the veterinary diagnostic laboratory *IDEXX*. All cases were either diagnosed with blood glucose over 15 mmol/L; 8 were newly diagnosed, 2 were euthanased at diagnosis, 11 were on insulin <1.5 U/kg and 4 had unknown insulin treatment histories. Veterinarians caring for cats were contacted and the history, clinical details and therapeutic history of the cases were obtained to ensure these cats did not have other forms of diabetes, such as acromegaly. Further, 14/20 cats had fPLI <12 μg/L, 24/24 had normal total TT4. On haematology and biochemistry (and urinalysis where available), biochemical evidence of renal dysfunction was present in 6 of 26, liver disease in 7, renal and liver disease in 5, and there were no data for 1 animal.

ABB control cats in cohort two (*n* = 47) were eight years or older, with a screening blood glucose concentration (measured after entry to the clinic and any time in relation to eating) of <8.1 mmol/L measured from fluoride-oxalate samples on an automated serum chemistry analyser (*n* = 42 from *IDDEX*), or immediately after sample collection using a handheld glucose meter calibrated for cats (Abbott AlphaTRAK) (*n* = 5 from research projects) [28]. Of these, 14 were male, 33 female. Screening blood glucose concentration <8.1 mmol/L is lower than the upper limit of the screening blood glucose reference range (9.7 mmol/L) and in 45/47 cats, screening blood glucose was <6.5 mmol/L, a stringent cut-off point for aged, client-owned cats that have not been fasted and are potentially subject to stress (which may cause hyperglycaemia) [29]. Burmese cats without signs of diabetes have been shown to have fasting blood glucose concentrations on average 1.9 mmol/L higher (*p* < 0.05) than age-matched domestic cats [5]. Of these 47 cats, one had no haematology, biochemistry or urinalysis data available, evidence of renal dysfunction was present in 15, liver disease in 2, liver and renal disease in 6, and 23 had normal haematology and biochemistry (and urinalysis when available) findings.

### 2.2. US-Bred Burmese Cats

A third cohort comprised 84 DNA samples from American-bred Burmese cats; 37 female, 21 male and 26 of unknown sex. These cats were not tested for diabetes, but are representative of a population with a normal low risk of type 2 diabetes (~0.25% to 1%) [17].

### 2.3. Genome-Wide Association Mapping and Analyses for Association with Diabetes: Genomic

Genomic DNA was extracted using a QIAamp DNA Blood Mini Kit (QIAGEN, Hilde, Germany). Genotyping in the first cohort of cats was performed using the Illumina iSelect custom feline genome chip containing 58,444 SNPs. All SNPs were analysed with Illumina Genome Studio software and subjected to stringent quality control procedures. SNPs with GenTrain Scores <0.60, call rate <0.95, or failed genotype concordance in replicates were removed from analysis. SNPs were excluded if they exhibited a minor allele frequency (MAF) in cases <0.01 due to sample size, or case/control bias in missingness. In total, 38,487 SNPs remained after these cut offs. Association testing was performed in PLINK [30] using Fisher’s Exact test, with *p* values corrected for multiple tests by Bonferroni correction. Correction for genetic admixture (caused by potential misrecorded matings) was assessed and corrected using Eigensoft [30]. The initial screening GWAS, with 10 cases and 10 controls, was regarded primarily as an exploratory study, with any suggestive findings to be followed in a subsequent cohort.

### 2.4. PCR, Gel, Digest and High-Resolution Melt Analysis

Targeted genotyping assays were developed to analyse the novel variants that we identified. Depending on the SNP, assays used were allele-specific PCR, single-strand conformation polymorphism PCR, restriction digestion or high-resolution melt analyses. Primer sequences are in Supplementary Table S1.

In general, 20 ng of genomic DNA from each sample was amplified by PCR in a 20 µL total PCR reaction mixture including 0.2 U Platinum Taq DNA polymerase (Life Technologies, Perth, Australia), 50 mM MgCl_2_ (Life Technologies, Victoria, Australia), 10 mM nucleoside triphosphates (dNTPs) (SIGMA-Aldrich, Castle Hill, Australia) and 50 ng primers (SIGMA-Aldrich, Castle Hill, Australia). The cycling parameters were: one cycle at 96 °C for 5 min, then 34 cycles of 96 °C for 20 s, 58–66 °C for 20 s, 72 °C for 30 s and a final extension at 72 °C for 3 min. The PCR products were separated on a 3% agarose gel (BIO-RAD Laboratories, Hercules, CA, USA).

Genotyping was performed where possible by differential restriction enzyme digestion. After digesting 15 μL of PCR product for 3 h with the appropriate enzyme (Table 2), and manufacturers’ instructions, the products were electrophoresed on a 3% agarose gel.

If PCR products (such as SNP6, chrC2:84361252+84361412), could not be distinguished by gel separation, they were tested by High Resolution Melt analysis. Briefly, they were amplified from 20 ng of genomic DNA in a 20 µL total PCR reaction mixture containing ResoLight Dye (Roche, Indianapolis, IN, USA), 0.2 U Platinum Taq DNA polymerase (Life Technologies, Victoria, Australia), 50 mM MgCl_2_ (Life Technologies, Victoria, Australia), 10 mM dNTPs (SIGMA-Aldrich, Castle Hill, Australia) and 50 ng primer (SIGMA-Aldrich, Castle Hill, Australia). Following PCR, the samples were heated to 95 °C, rapidly cooled to 45 °C, reheated to 70–95 °C with a ramp rate of 0.2 °C/s, and analysis of melt curves was conducted using the Light Cycler 480 Software Release 1.5.0 SP3 (Roche, Sydney, Australia) [31].

Allele-specific PCRs were used to detect the G/A polymorphism in the *E2F6* gene. The allele-specific reaction mixture contained 20 μg of genomic DNA in a 20 µL PCR reaction mixture including 0.2 U Platinum Taq DNA polymerase (Life Technologies, Victoria, Australia), 40 mM MgCl_2_ (Life Technologies, Victoria, Australia), 10 mM dNTPs (SIGMA-Aldrich, Castle Hill, Australia) and 50 ng allele specific primers (SIGMA-Aldrich, Castle Hill, Australia). Primer sequences are indicated in Supplementary Table S1. Forward primers were designed with a T-A nucleotide mismatch to create primer 3′ instability and increased allele specificity. The G and A 3′-terminal nucleotides of the allele-specific forward primers annealed to the G and A nucleotides of the *E2F6* gene. The reverse primer was designed to anneal to a highly conserved region of the *E2F6* gene in Australian-bred Burmese. The thermal profile consisted of one cycle at 96 °C for 5 min, then 34 cycles of 96 °C for 20 s, 58–66 °C for 20 s, 72 °C for 30 s, and a final extension at 72 °C for 3 min. Reactions were performed to determine AA/AG/GG status of all ABB DNA samples. The PCR products were separated on a 3% agarose gel (BIO-RAD Laboratories, Hercules, CA, USA).

### 2.5. Sequencing of Candidate Genes

Genes near the SNPs identified in the GWAS as loci of interest (Table 1) were inspected using the November 2017 ICGSC Felis_catus 9.0/felCat9 and candidate genes were chosen for sequencing On chromosome A3, SLC8A1 located within 20 Kb of SNP1 and E2F6 was within 20 Kb of SNP2. On chromosome C2, SNP4 was located adjacent to 3 candidate genes; ETV5 within 20 Kb, DGKG within 200 Kb, and TRA2B within 150 Kb. On chromosome C2, SNPs 5–6 were located near IGF2BP2 within 200 Kb, and LIPH within 100 Kb.

Exons and the 5′ and 3′ untranslated regions (UTR) of all candidate genes were sequenced (primer sequences shown in Supplementary Table S2). To amplify the DGKG gene, M13 tails were added to all primers, and a touchdown PCR protocol was followed [32]. Sequencing for the DGKG gene was performed with Version 1.1 big dye terminator sequencing using M13 forward and reverse primers [33]; the remaining genes sequenced with the PCR primers. PCR products were cleaned using the PCR Clean up Kit (Qiagen), 20 ng/μL of this product was then mixed with 20 ng/μL of the forward or reverse primer (Sigma-Aldrich, Castle Hill, Australia), Big Dye Terminator (V1.3) and Buffer (×5) (Ambion). The cycling parameters were: one cycle at 96 °C for 3 min, then 25 cycles of 96 °C for 10 s, 50 °C for 2 s, 60 °C for 4 min. The sequencing reactions were cleaned and analysed on the ABI3730. Sequences were analysed using Sequence Scanner 2 software (Applied Biosystems, Victoria, Australia). Sequences were aligned using Clustal Omega 2019 (EMBL-EBI, Cambridge, UK) to identify SNPs. Variant protein sequences were submitted to Protein Variation Effect Analyser (PROVEAN V1.0) (Rockville, MD, USA) protein prediction software [34]. No further analysis was conducted on SNPs that did not result in protein coding changes.

## 3. Results

### 3.1. Search for Genetic Markers Associated with Type 2 Diabetes

Our strategy was to conduct an exploratory GWAS with 10 diabetic and 10 control ABB cats to characterise sequence polymorphisms between diabetic and non-diabetic Burmese which might be examined in a larger population sample, and to validate any candidate polymorphisms in a larger cohort. Genotyping was performed using custom Illumina arrays [35]. As expected for a sample this small, no SNP provided a *p* value below the threshold for significance after Bonferroni correction (i.e., *p* 8 × 10^−7^). However, six SNPs were identified with promising *p* values <0.0005 (Table 1). These SNPs tagged regions on cat chromosomes A3, C1 and C2. The SNPs on the custom Illumina chip had custom numerical annotations. For convenience, these six candidate SNPs are designated here as SNPs 1 through 6.

### 3.2. Validation of the Candidate Type 2 Diabetes Associated SNPs

The six candidate SNPs were genotyped in a second cohort of ABB cats supplemented with samples from Burmese cats from the low-prevalence US population. Only one (SNP6) was not significant in the validation cohort. SNPs 1–4 demonstrated significant association after Bonferroni correction while a further SNP (SNP5) had a *p* value of 0.01 (Table 2).

Haplotype analyses demonstrated that SNPs on chromosome C2 tagged intervals that were strongly associated with type 2 diabetes (Table 3). Even the SNP that was not significant as a singleton (SNP6), demonstrated a significant association when combined with SNP5, tagging a chromosome interval that showed association. As these SNPs span a genomic interval of well over 500 kb, these results suggest that they may tag a founder haplotype that increases the risk of type 2 diabetes, or that the individual variants near these SNPs interact to increase susceptibility.

### 3.3. Selection of Candidate Type 2 Diabetes Genes

Genes near the associated SNPs were inspected using the November 2017 ICGSC Felis_catus 9.0/felCat9. The two associated SNPs on chromosome A3, SNP1 and SNP2, were located within introns of *SLC8A1* and *E2F6*, respectively. There are no other known genes within 50 Kb 5′ or 3′ of these SNPs. Neither of these genes has previously been implicated in type 2 diabetes susceptibility. The most highly associated SNP, SNP3, resided in an area with no known coding genes within at least 300 Kb.

SNP4 is located in an intron of *ETV5*. The human orthologue of *ETV5* was reported to be associated with type 2 diabetes and obesity in several human populations [36,37]. SNPs 5 and 6 are within the same intron of the *LIPH* gene, which encodes a membrane-bound triglyceride lipase. The genomic region on chromosome C2 located between SNPs 4 and 6, contains only four other known genes based on sequence homology or conserved synteny with other species. These are: *TRA2B*, *CHCHD4*, *IGF2BP2* and *SENP2*. Of these, *TRA2B* (also known as SFRS10) has been linked in humans and mice to obesity and so to related metabolic syndromes including type 2 diabetes [38]. Studies have indicated the involvement of *IGF2BP2* in type 2 diabetes in humans [39].

### 3.4. Search for Polymorphisms within Candidate Genes

To identify genetic variants in the genes identified above, all coding regions of these genes were sequenced, including the 3′ and 5′ UTRs of each. Information about the genomic regions sequenced, any discovered SNPs, the nucleotide position of each SNP, any predicted amino acid change and the effect of the mutation as estimated by the PROVEAN algorithm is shown in Supplementary Table S3. Association results of selected SNPs with the diabetes trait are shown in Supplementary Table S4. Eleven SNPs were discovered in *SLC8A1*, of which two were found to contain a potentially deleterious amino acid substitution. Twelve SNPs were identified in *E2F6*, with one predicted to be deleterious. However, none of these SNPs were significantly associated with the disease phenotype, suggesting that they were commonly occurring in the Burmese cat breed but not the cat(s) used to establish the genome sequence of this species. No likely causal mutations, including non-synonymous substitutions, polymorphisms in splice junctions, introduction of premature stop codons or insertions/deletions resulting in frame-shifts were found in either *IGF2BP2* or *DGKG*. Fourteen SNPs were identified in *ETV5* with two predicted to be deleterious, but these also were not significantly associated with the disease phenotype. Two SNPs and one deletion were identified in the *TRAF2B* gene; all were found to reside within introns.

Finally, eight novel SNPs were identified in *LIPH*, none of which were in exons. One of these SNPs was significantly (*p* = 1.6 × 10^−5^) associated with diabetes, conferring a relative risk of 2.6. No feline transcripts of this gene have been sequenced, but by alignment of the cat genomic and human cDNA sequences, the significant SNP is located in the presumed 3’UTR region. This is reminiscent of the 3’UTR SNP in the *IL12B* gene which affects gene expression and is associated with susceptibility to type 1 diabetes and other diseases in humans [40,41]. This novel SNP was confirmed and was significantly associated with type 2 diabetes in the total cohort of ABB cats.

## 4. Discussion

Our exploratory GWAS found an association of six SNPs with type 2 diabetes. Seven candidate genes were investigated for the presence of variants that could contribute to type 2 diabetes. Of these candidates examined, only *LIPH* exhibited a novel disease-associated variant. Two genes near SNP 4, *ETV5* and *DGKD*, did not have disease-associated sequence variants. The region on Chr C2 near SNPs 5 and 6, contained four known genes (*TRA2B*, *CHCHD4*, *IGF2BP2* and *SENP2*) but none of these genes contained disease-associated protein-coding changes, despite *TRA2B* and *IGF2BP2* having association with type 2 diabetes in other species [39]. Thus, these gene variants compared to the reference cat genome sequence are found in the Burmese cat lineage but are not associated with T2D susceptibility.

SNP3, the most highly associated SNP, is in a genomic region of low gene density which has no known coding genes within at least 300 Kb. Many SNPs associated with disease risk occur in such “gene deserts”. They may reside in enhancer elements [42] and regulate expression of distant genes, as shown for type 1 diabetes risk genes [43].

This study demonstrates the potential of ABB cats to identify novel genes involved in type 2 diabetes susceptibility. The ABB cat population originated from a small number of founders, with subsequent inbreeding leading to extensive linkage disequilibrium (LD) [44]. In US-bred Burmese cats, a more outbred population, average haplotype blocks have been reported to be 0.5 Mb [44], along with an average inbreeding coefficient of 0.22 and observed heterozygosity of 0.4 [45]. Extended LD observed in many domesticated animal breeds can be created by population bottlenecks, small effective population sizes and use of popular sires, and has proved highly beneficial in genetic mapping [45]. Thus, in this study, the ABB cat, with its genetic architecture and high prevalence of type 2 diabetes, allowed identification of genetic elements of large effect size using smaller sample sizes than required for outbred populations.

Up to 7% of Australian cats seen by veterinarians are Burmese [12], and up to 10% of ABB cats over 8 years old develop diabetes [5], so ABB cats comprise a significant proportion of the population of domesticated cats requiring veterinary care. Diabetes has major quality of life implications for cats and also major cost implications for owners [46]. Our results have clinical relevance for Burmese cats at increased risk of diabetes in Australia, and in other countries like the UK [14] and New Zealand [15], and possibly for other domestic cat populations.

Our results could reduce the burden of care on ABB cat owners by identifying cats carrying risk alleles. Identifying at-risk cats allows early intervention to identify pre-diabetic cats and prevent development of overt diabetes. Early identification of pre-diabetic cats is important, once cats are pre-diabetes (fasting blood glucose >7.5 mmol/L and 3 h glucose >14 mmol/L), they have an 88% probability of being diabetic within 9 months [47]. Potential interventions include weight control, a low carbohydrate diet to reduce postprandial increases in glycaemia, and insulin-sensitising drugs. Currently such drugs are not commonly used because of the difficulty in identifying at-risk pre-diabetic cats. Further, general dietary and body weight recommendations for cats are not always adopted by owners but may have increased uptake if owners knew their cat was genetically at risk. Identification of pre-diabetic and subclinical diabetic cats could result in these cats being managed without the need for insulin. Further, improved monitoring would expedite early diagnosis of diabetes and quick implementation of tight glycaemic control. This has benefits for diabetic cats and their owners because remission rates in excess of 80% can be achieved compared to 30–40% remission rates if tight glycaemic control is delayed [48].

This study, by identifying risk loci in the ABB cat population, particularly allows cat breeders to make informed choices to avoid type 2 diabetes risk in their breeding pedigrees. Cats are bred between 1 and 6 years, well before clinical signs of diabetes are evident, so identification of carriers of risk loci would help reduce type 2 diabetes prevalence. Further investigations may also show whether these loci may also contribute to diabetes in other cat breeds.

Type 2 diabetes in ABB cats is clearly not monogenic; while we have mapped a few loci, their susceptibility alleles are not present in all diabetic cats genotyped, and doubtless others with smaller effects are also involved. Despite this, our identification of risk alleles is a step toward a greater understanding of the metabolic basis of diabetes in cats. Identification of risk alleles in cats may also have relevance for the human disease [49], and this cat population could provide an animal model for studying the role of these genes in the disease process.

## Figures and Tables

**Table 1:** Candidate SNPs identified from the exploratory GWAS. A1 indicates the minor allele (i.e., least frequently observed based on the whole sample); F_A and F_U indicate the frequency of the minor allele in the affected and unaffected cats, respectively. The *p* value is shown calculated by Fisher’s Exact test and Bonferroni corrected by PLINK. No odds ratio could be calculated for SNP2 because the associated allele was not found in unaffected cats. SNPs were named in the Illumina array only as custom numbers; for convenience, they are named here as SNPs 1 to 6.

SNP	Illumina No.	A1	F_A	F_U	*p*	Odds Ratio
SNP1	10566	G	0.75	0.15	0.00014	17
SNP2	10762	G	0.5	0	0.00026	NA
SNP3	30747	G	0.1	0.65	0.00033	0.059
SNP4	36147	G	0.8	0.2	0.00015	16
SNP5	36165	A	0.1	0.65	0.00033	0.059
SNP6	36166	A	0.1	0.65	0.00033	0.059

**Table 2:** Testing candidate SNPs in a validation cohort. DNA samples from a further 37 diabetic ABB cats, 47 normoglycaemic Australian-bred Burmese cats, and 84 Burmese cats from the low-prevalence US population were genotyped at the SNPs implicated by the GWAS. Results were analysed using Plink. A1 indicates the minor allele (i.e., least frequently observed based on the whole sample); F_A and F_U indicate the frequency of the minor allele in the affected and unaffected cats, respectively. The *p* value is shown calculated using Fisher’s Exact test and Bonferroni correction. No odds ratio could be calculated for SNP2 because the associated allele was not found in the non-diabetic cats.

SNP	A1	F_A	F_U	*p* Value	OR	Chromosome Position
SNP1	T	0.59	0.38	0.00089	2.4	chrA3: 10995614
SNP2	G	0.29	0.52	0.00043	0.4	chrA3: 134626291
SNP3	C	0.17	0.46	5.35 × 10^−6^	0.2	chrC1: 23237623
SNP4	C	0.63	0.41	0.0013	2.5	chrC2: 83660325
SNP5	T	0.32	0.49	0.012	0.5	chrC2: 84129862
SNP6	A	0.30	0.34	0.53	0.8	chrC2: 84135537

**Table 3:** **Haplotype analyses**. Data from the validation cohort were analysed using the haplotype association test implemented in PLINK. Results for the most associated haplotype are shown.

Chr	SNPs	Haplotype	F (Diabetic)	F (Unaffected)	*p* Value	Chromosome Position
C2	4 and 5	CC	0.51	0.29	0.0006	93563976 and 94157910
C2	5 and 6	CC	0.55	0.34	0.001	94157910 and 94165023

## Supplementary Materials

The following are available online at https://www.mdpi.com/2073-4425/11/11/1369/s1. Supplementary Table S1: Nucleotide sequence of primers used for genotyping candidate SNPs, Supplementary Table S2: Primers used to amplify and sequence fragments of candidate genes, Supplementary Table S3: Search for polymorphisms within candidate genes, and Supplementary Table S4: Testing Novel SNPs for Asociation with Type 2 Diabetes.
