# Supplementary material for: Mapping of Diabetes Susceptibility Loci in a Domestic Cat Breed with an Unusually High Incidence of Diabetes Mellitus
Source: Genes (Basel). 2020 Nov 19;11(11):1369. doi: 10.3390/genes11111369 (PMC7699364; doi:10.3390/genes11111369)
Supplement: Supplementary file 1 [file genes-11-01369-s001.pdf]

SUUPLEMENTARY MATERIAL

Article

Mapping of Diabetes Susceptibility Loci in a Domestic Cat Breed with an Unusually High Incidence of Diabetes Mellitus

Lois Balmer [1,2,†], Caroline Ann O'Leary [3,†], Marilyn Menotti-Raymond [4], Victor David [5], Stephen O'Brien [6,7], Belinda Penglis [8], Sher Hendrickson [9], Mia Reeves-Johnson [3], Susan Gottlieb [3], Linda Fleeman [10], Dianne Vankan [3], Jacquie Rand [3,11,‡] and Grant Morahan [1,*,‡]

[1] Centre for Diabetes Research, Harry Perkins Institute for Medical Research, University of Western Australia, Nedlands 6009, Australia; l.balmer@ecu.edu.au
[2] School of Medical and Health Sciences, Edith Cowan University, Joondalup, Perth 6027, Australia
[3] School of Veterinary Science, the University of Queensland, Gottan 4343 , Australia; c.oleary@uq.edu.au (C.A.O.); m.reevejohnson@uq.edu.au (M.R.-J.); susanalisongottlieb@gmail.com (S.G.); d.vankan@uq.edu.au (D.V.); j.rand@uq.edu.au (J.R.)
[4] Laboratory of Genomic Diversity, Center for Cancer Research (FNLCR), Frederick, MD 21702, USA; Marilyn.Menotti@gmail.com
[5] Laboratory of Basic Research, Center for Cancer Research (FNLCR), National Cancer Institute, Frederick, MD 21702, USA; davidvic@mail.nih.gov
[6] Laboratory of Genomics Diversity, Center for Computer Technologies, ITMO University, 197101 St. Petersburg, Russian Federation; lgdchief@gmail.com
[7] Guy Harvey Oceanographic Center, Halmos College of Arts and Sciences, Nova Southeastern University, Ft Lauderdale, FL 33004, USA
[8] *IDEXX* Laboratories East Brisbane, Queensland 4169, Australia; belinda.penglis@bigpond.com
[9] Department of Biology, Shepherd University, Shepherdstown, WV 25443, USA; shendric@shepherd.edu
[10] Animal Diabetes Australia, Melbourne, Victoria 3155, Australia; l.fleeman@AnimalDiabetesAustralia.com.au
[11] American College of Veterinary Internal Medicine, University of Zurich, 8006 Zurich, Switzerland
* Correspondence: grant.morahan@perkins.org.au
† These authors contributed equally to this work.
‡ Joint senior authors.

Supplementary Table 1. Nucleotide Sequence of Primers used for Genotyping Candidate SNPs.

All sequences are listed from 5' to 3'. SNPs are listed as specified on the original Illumina chip used for genotyping, annotated with chromosome number and base position from the cat assembly. The genotyping method was performed by differential digest of restriction enzymes shown, or by allele specific PCR (ASP, where the allelic SNP is shown in lower case in the forward primer) or by high resolution melting (HRM).

SNP Name	Primer sequences		Chromosome Position	Amplicon Size (bp)	Alleles	Method
SNP1	CGTGTGCTTGGCATATATCAG	CCAAAAACTCTGGGAACTGC	chrA3:109955841+109956008	168	T/C	HpyCH4V
SNP2	AAACTCCGACTCCTCGTGTa	GCAAGAGACAACGTGCAAGG	chrA3:134626269+134626401	133	A/G	ASP
SNP3	GCCCCAGATATGTCAAGACC	CGTGACAAGCATTGTTCCTG	chrC1:23237549+23237718	170	T/C	Aci1
SNP4	ACATTCCAGGGAGTCGTGAG	GCTGGCTGTACACAGGATTG	chrC2:83660239+83660383	145	T/C	Bsr1
SNP5	CTGGCCCAGAACCTAGGAG	CTCTGGAAAAGCCCATTGTG	chrC2:84129732+84129896	165	T/C	Bcc1
SNP6	GAAAAGCCTAAGATTTACTGACCAG	GGGGCAATTCATGAGACAGATC	chrC2:84135455+84135615	183	A/C	HRM
SNP7	GGGTCACATGCATTCAGCTA	CATAAGAGCTGATAATGAGGGT	chrD2:88458324+88458478	155	G/A	HRM

Supplementary Table 2. Primers used to amplify and sequence fragments of candidate genes.

The number following the primer name (SLC8A1-*) indicates a combined exon/intron region, this may often encompass more than one exon and/or intron; the initial and final number may also include some 5' and 3' untranslated region, respectively.

Primer Name	Primer sequences		Chr. Position	Amplicon Size (bp)
**E2f6 promoter**	TAGCAAGGGGTGAGCATTTC	AACTTGGATGCGAGAAAAGC	chrA3:134624551+134624868	318
**E2f6-1**	CCCCAGGGAGAATAAAAACC	CCCACAACCTCACAAGTGTTT	chrA3:134624873+134625193	321
**E2f6-2**	ATTTCGATGGGTGTCTATGC	ACTTATGTACCTTGTGCGAC	chrA3:134628717+134629223	507
E2f6-3	CTAACTTGCGTCTTCACCTC	TTAAAGTAACCTCTGCCTTGG	chrA3:134630659+134630990	332
E2f6-4	TCCGGAAATTGTCACCATAG	CAAGCACATACTCTGGAGAC	chrA3:134632242+134632940	699
E2f6-5	CAGTATCCCTTGAATTGGCC	GGCTAAACTACCTTGTCGTC	chrA3:134635371+134635984	614
E2f6-6	CTGTGCCATTGACAGAACG	TGGAAATGTGGGACTAGAGC	chrA3:134636168+134637350	1183
**ETV5-1**	CTCGGACCAATGAGAACCAG	TAGAAAGTGCACCCCGACTT	chrC2:83644016+83644397	382
**ETV5-2**	TATAGGGAGCGGTTAGTAGC	AGTTTTCCACGCATGGATGT	chrC2:83647112+83648153	1042
**ETV5-3**	CCAGTTCCAGGGTTTGCAG	ATCCCTCCCTAGAAGCCAC	chrC2:83672298+83672793	496
**ETV5-4**	AACGTGGACTGCAACCTAGC	GGAGTGCCTCAAGAACAGGT	chrC2:83673335+83673897	563
**ETV5-5**	GGAAGAGGGTGCCTGAACTT	TGAAGCTAGGGGCAGGACTA	chrC2:83685232+83685933	702
**ETV5-6**	TGAATGTGACCATCCGCTG	GGAAGCTGAGCATGTTAAGC	chrC2:83687181+83687722	542
**ETV5-7**	CTTCCCCGCAGTCTTAGCTC	AAGAACTGCCGAGCCCATTG	chrC2:83691970+83692781	812
**ETV5-8**	CACCTCTTAGCAGTTGTGAC	GTTTCTGTGTACCCAAGAGC	chrC2:83696772+83697288	517
**ETV5-9**	CCCCATGCCCTGTTCCAG	GCCTGTTTCTTCCGCTCAC	chrC2:83702021+83703906	1886
**Liph-1**	TCGACCTTCCACTGAAAAGAG	CTGAAGGGAGTGGGCTGATA	chrC2:84116297+84116638	342
**Liph-2**	CTAGATCCAGCCTCCTGCTG	AGTGCACAACTTGCACAACC	chrC2:84134561+84135238	678
**Liph-3**	GGCCTCAGTTTCCTCATCTG	AAGGAAGATCGCTGTTTGGA	chrC2:84136081+84136717	637
**Liph-4**	TTATCCCCATGTCCCTGGTC	TCCACTCCAGCAAGACATCC	chrC2:84142106+84142704	599
**Liph-5**	ACTGACCCAAACTCCCTTAC	GGGTTGCTTCCATATCTTCG	hrC2:84143918+84144467	550
**Liph-6**	TAAGCTGAGTCTCAATGTGC	GCTTGGAAACTCTTTGGAAC	chrC2:84147098+84147785	688
**Liph-7**	TGACAACTGGAGACCACTGG	ATCCTTCACAGCAAGCAAGG	chrC2:84150491+84151162	672
**Liph-8**	GCATGCCTCTCTTGTGCC	CTTGCAAGCAGTGTGGGTG	chrC2:84159419+84160060	642
**Liph-9**	GTTGTTGATGTGGGCTATGC	AGTTTGAGACAGCCAGAGTG	chrC2:84161025+84162370	1346
**SLC8A1-1**	CCACACGGAGAATCCTTCAG	CGCAAGGCAGAACCCTAAC	chrA3:109917045+109917340	296
**SLC8A1-2a**	GGATCTGGCTCTGATGGTGT	GTCTTGGAAGATGGCCTGTC	chrA3:109937002+109937953	952
**SLC8A1-2b**	TCGCTTGGGTAGCAGATAGG	ATGCATACGCTCTCCCATTC	chrA3:109937836+109938967	1132
SLC8A1-3	GGAGCTATGCATTGGGTGAC	GAAGAGTGCAGGCATCCAG	chrA3:110181615+110181989	375
SLC8A1-4	TGCCTGGATTTCTTTCTGC	ATGTGCCACGGCTAAATG	chrA3:110184954+110185758	805
**SLC8A1-5**	TCTGGTAGCAAACCAGTGC	TTGATGACCCACCATACTCC	chrA3:110189456+110189753	298
**SLC8A1-6**	CTCCGGCCTTATGATCTCAC	TGAGCCAGTTCCTGACATTG	chrA3:110194028+110194389	362
**SLC8A1-7**	TGAGCCTAACATCCCCTACC	TTTACCCTGGAAGGTCGTTG	chrA3:110198110+110198436	327
**SLC8A1-8**	CCACTGTGAGGGGGTATGTC	AGGTCAGGAAGGCACATGG	chrA3:110221496+110222022	527
**SLC8A1-9**	GAAGCTTCCAGTGCTCCAC	CTTGAAGCTGGGTTCCATC	chrA3:110244193+110244768	576
Traf2b-1	GAGCGCTTCGGTACTCAGC	TTCCCAGCCTCAGACTTCG	chrC2:83775198+83775481	284
Traf2b-2	AGTTTCAGTTTTGGCACTGG	CTGGAAGGCACTTCTTTGC	chrC2:83781476+83781927	452
Traf2b-3	TTATCTTGGAAGGGGATTGC	GCACACACCAATTTCAAAGC	chrC2:83786698+83787000	303
Traf2b-4	TGGAAGAGAGCAAGTAGTGG	CCAAGAGTGTTTTGATGAGG	chrC2:83787790+83788383	594
Traf2b-5	CTGCCCATTCTTAATGCTTG	GTCAGGACTGGATGAAATGC	chrC2:83788641+83789399	759
Traf2b-6	AAAGGGTGGCAAATGTAGC	CACAACTGAGGGGCAGTAG	chrC2:83792358+83793305	948
Traf2b-7	TCCCTGTAGAGAATCTGGTG	TTTAGCCCAAGGGATACTG	chrC2:83793562+83794786	1225
Traf2b-8	AAATAAGCCATGCTGCTGCT	TGTTGCAACTGATTCAGGAGA	chrC2:83790344+83790615	272

DGKG_ex1	CCCTGGTGAACAAAACTCCA	CTGTCAGGCCCACTCTCATT	chrC2:83451030+83451269	240
DGKG_ex2	AGTCCTGGGAATGAGGGATT	ATAATTCCAGGGAGCACCAA	chrC2:83463397+83463728	332
DGKG_ex3	GTCCCTTTGCTTCTCTGCAC	TCTCATTTCCACCCCGTAAG	chrC2:83471640+83471979	340
DGKG_ex4	ACAGTCAGCCCCAACAAATC	ATGAACCCGCATCAAGACTC	chrC2:83472289+83472699	411
DGKG_ex5	AATGGGCTCCATTCAGAGAG	AAGCAGGCATCACCAGTACC	chrC2:83480505+83480975	471
DGKG_ex6	GGGTAACCTGGCTGCAATAA	TTTTGTGATCCTGGGCTTCT	chrC2:83485433+83485849	417
DGKG_ex7	CTGGGGTTGGATTTTGAGAA	ACTCCGGTGGAAAGGTTAGG	chrC2:83489427+83489795	369
DGKG_ex8	GCAGAACCCAGAGAGGAGTG	TTTTGGGCTGGCCTAGAGTA	chrC2:83490008+83490495	488
DGKG_ex9	GCCAACCATGTAGGAGAGGT	GCACCTAGACTCTGCCTGGA	chrC2:83494723+83495185	463
DGKG_ex10	GAGGTGGGCAGATGGTTAGA	CCCACTAGCCTCCACAGTTC	chrC2:83496580+83496987	408
DGKG_ex11	GAGCAGCCCCATATCCACTA	GGTGATTCACTGGAGGCACT	chrC2:83499628+83500063	436
DGKG_ex12	TCTGAACCCTCACCCTTGAC	GGCAATGACCGTTCAAACTT	chrC2:83500816+83501116	301
DGKG_ex13	GATCCAAAGCCCTGTTTTGA	ACCTCCCCTTTTCACCACTT	chrC2:83502554+83502979	426
DGKG_ex14	CCTGCTGGCTGAAAAAGAAC	ACCAAGGCACAGGGATGTTA	chrC2:83505118+83505418	301
DGKG_ex15	TCTTCTCCCCTCTTGGGTTT	AGGGTAAAATGGCAGGAAGG	chrC2:83506251+83506590	340
DGKG_ex16	TCAGGGCCGTAACATACTCC	CCCAAGAGTGGAAACAAGGA	chrC2:83509323+83509641	319
DGKG_ex17	CCCAAAGGACAGAATTTGGA	GAGCATCCCTTGAAATTGGA	chrC2:83513632+83514050	419
DGKG_ex18	GGCTTCCAAATCTCCCATTT	GGGGACAACAGAAGACCAGA	chrC2:83516074+83516533	460
DGKG_ex19	TAGCCCCTAACCTTGGACCT	TCTGCTTGGAAATGGAAAC	chrC2:83525506+83525931	426
DGKG_ex20	TCTGAGTTCTTTGCCGGAAC	TAGCGGTGTTTGGGAATAGG	chrC2:83554394+83554820	427
DGKG_ex21	CCTTGGGTTGGGATACACTG	TTTGCTAATAGGCCCACCTG	chrC2:83575476+83575787	312
DGKG_ex22	TCTGACCACAACCATCCTGA	GCTTCTGTCCGTCACTCTCC	chrC2:83597003+83597305	303
DGKG_ex23	AAGTGGGCTCAGCAATCTGT	GGTACCTGAATGGGTGGATG	chrC2:83599419+83599806	388
DGKG_ex24	TGTCTCTCCCCAGTGTTTCC	TTGGTTTAACTGGCATTTGG	chrC2:83609641+83609990	350
IGFBP2_ex1	GGAGGAGGAGGAGAGACGA	CGAGAGTTGAGGGTCTGGTG	chrC2:83905414+83905827	414
IGFBP2_ex2	GTTAGCGGAATGGAGAGGTG	CCCCAATAAGGCACCAGATA	chrC2:83906895+83907534	640
IGFBP2_ex3	GGTCAGTTGCAGGACCATCT	AGACAGAACAGGAGGGCTCA	chrC2:84019892+84020545	654
IGFBP2_ex4	AAAAACAAACTGCCCCAATG	TTGGAGTTGACCCAGCCTAT	chrC2:84020845+84021518	674
IGFBP2_ex5	TGGCTTGTCAAAAATCATGG	CACAGGTCCCACCTCAGTTT	chrC2:84024654+84025277	624
IGFBP2_ex6	ATCAGCTCTGAGGGAGCAAA	CACTGTGAAGTCCCCAGCTT	chrC2:84026875+84027492	618
IGFBP2_ex8	TCTGGCTGGTGTTCTTCCTT	ACTGGATTCCCTCACCACAC	chrC2:84037691+84038377	687
IGFBP2_ex9	GGCAAGTATTTGATGCGACA	GCCTTCCATTTTGCTCCATA	chrC2:84038336+84038955	620
IGFBP2_ex10	GGGCAACTACACAGGCAAGT	AACCTCTGGGTGCTGAGCTA	chrC2:84040387+84041058	672
IGFBP2_ex11	ATTTGCTGGCCGTGAATAAC	TGCCACCTGTGGATTTGTAA	chrC2:84051591+84052225	635
IGFBP2_ex12	GCACCATTTTGACCATTTCC	GATTAATCTTGGGGGCCAAT	chrC2:84052746+84053151	406
IGFBP2_ex13	GAACTCCTTGCACTGCTTCC	AAATGCATCCGACCAAACTC	chrC2:84056145+84056830	686
IGFBP2_ex14	ACAGCCAGGTCTCTGAAGGA	GGGGAGAGTGTAGGACACCA	chrC2:84057657+84058310	654
IGFBP2_ex15	GGCAGGGATGAAAATGAAGA	TGAGATGTGCTTGGCTTGAC	chrC2:84059224+84059919	696
IGFBP2_ex16	GCGGGTAGACAACAGTTTCC	TTCCTCAGGATGGTCTTTGG	chrC2:84061175+84061522	348

Supplementary Table 3 Search for Polymorphisms within Candidate Genes.

"Amplicon" refers to the number of the amplicon of genes for which multiple exons were amplified and sequenced. Sequences were analysed using Sequence Scanner 2 software (Applied Biosystems, Victoria, Australia). Sequences with SNPs identified were aligned using ClustalW2 (EMBL-EBI, United Kingdom). Protein sequences encoded by missense SNPs in were submitted to the Protein Variation Effect Analyzer (PROVEAN) protein prediction software (Rockville, MD, USA) (Choi Y, 2012) and the predicted Effect on protein function is shown.

"Status" indicates results from testing novel variants discovered by comparing sequences from ABB cats to the reference cat genome. Where the novel SNP allele was found in other ABB cats, both diabetic and non-diabetic, it is indicated as "Burmese SNP", a variant that may be specific to that cat breed. "T2D associated" indicates that the novel SNP allele was significantly over-represented in diabetic ABB cats compared to non-diabetic ABB cats.

Gene	Amplicon	Variant Position	SNP	Amino acid change	Effect	Position	Status
SLC8A1	1	**None Found**					
	2a	chrA3:109937274	C/T	no change		Exon	
	2a	chrA3:109937721	C/T	no change		Exon	
	2a	chrA3:109937722	T/C	W->R	Del -13.997	Exon	Burmese SNP
	2a	chrA3:109937809	T/C	F->S	Del -7.300	Exon	Burmese SNP
	2b	chrA3:109938205	C/T	no change		Exon	
	2b	chrA3:109938642	T/C	no change		Exon	
	3	chrA3:110181758	A/T			Intron	
	4	chrA3:110185140	C/T			Intron	
	4	chrA3:110185198	G/A			Intron	
	4	chrA3:110185500	C/T			Intron	
	5	chrA3:110189622	T/C			Intron	
	6	**None Found**					
	7	**None Found**					
	8	**None Found**					
	9	**None Found**					
E2F6	5'UTR	chrA3:134624635	A/G	no change		5'UTR	
	1	chrA3:134624897	A/G			Intron	
	2	**None Found**					
	3	chrA3:134630855	A/G	E->K	Del -2.529	Exon	Burmese SNP
	4	chrA3:134632649	A/G			Splice site	Burmese SNP
	4	chrA3:134632780	T/G			Intron	
	4	chrA3:134632849	T/C			Intron	
	4	chrA3:134632875	A/C			Intron	
	5	chrA3:134635794	G/T			Intron	
	5	chrA3:134635802	A/T			Intron	
	6	chrA3:134636471	A/G			Intron	
	6	chrA3:134636600	G/C	no change		Exon	Burmese SNP

	6	chrA3:134636631	T/C			3’ UTR	
ETV5	1	**None Found**					
	2	chrC2:83647185	A/G			Intron	
	2	chrC2:83647798	A/G			Intron	
	2	chrC2:83647821	CCCCA (Deletion)	no change		Intron	Burmese SNP
	3	**None Found**					
	4	chrC2:83673383	G/A			Intron	
	5	**None Found**					
	6	chrC2:83687387	G/A			Intron	
	6	chrC2:83687540	G/T			Intron	
	6	chrC2:83687690	C/T			Intron	
	7	chrC2:83692059	G/C			Intron	
	7	chrC2:8369228	C/T			Intron	
	7	chrC2:83692322	G/A			Intron	
	8	**None Found**					
	9	chrA2: 83702092	C/T	A->T	Del -5.000	Exon	Burmese SNP
	9	chrA2: 83702188	C/T	I->V	Neu -1.000	Exon	
	9	chrA2: 83702263	C/T	N->D	Del -6.000	Exon	Burmese SNP
	9	chrA2: 83702303	A/G			3’ UTR	
IGF2BP2	1	**None Found**				5'UTR	
	2	chrC2:84133261	C/G			Intron	
	3	**None Found**					
	4	chrC2:84247187	G/A			Intron	
	4	chrC2:84247191	G/A			Intron	
	4	chrC2:84247209	C/A			Intron	
	5	chrC2:84250826	T/delT			Intron	
	6	chrC2:84252889	A/G			Intron	
	6	chrC2:84253250	C/T			Intron	
	7	chrC2:84255023	C/T			Intron	
	7	chrC2:84255083	C/G	V->V		Exon	
	7	chrC2:84255259	C/T			Intron	
	7	chrC2:84255336	G/A			Intron	
	8	chrC2:84263856	G/A			Intron	
	8	chrC2:84266774	C/A			Intron	
	9	**None Found**					
	10	chrC2:84266774	C/A			Intron	
	11	chrC2:84277808	G/A			Intron	
	11	chrC2:84277866	G/A			Intron	
	11	chrC2:84277974	G/A			Intron	
	11	chrC2:84278137	G/A			Intron	
	12	chrC2:84278804	G/T			Intron	
	12	chrC2:84278806	G/A			Intron	

	12	chrC2:84278963	T/G			Intron	
	13	**None Found**					
	14	chrC2:84283914	C/T	H->H	Neu	Exon	
	15	**None Found**					
	16	chrC2:84287279	C/T			3’ UTR	
LIPH	1	chrC2:84116405	C/A			Promoter	
	1	chrC2:84116432	T/A			Pro/5’UTR	
	2	chrC2:84134822	G/T	L->F	Neu 0.822	Exon	
	3	**None Found**					
	4	**None Found**					
	5	**None Found**					
	6	**None Found**					
	7	**None Found**					
	8	chrC2:84159595	C/T			Splice site	Burmese SNP
	9	chrC2:84161272	G/A	no change		Exon	
	9	chrC2:84161404	C/T			Intron	
	9	chrC2:84161783	C/A			Intron	
	9	chrC2:84162155	C/T			3’ UTR	**T2D Associated**
DGKG	1	**None Found**					
	2	chrC2:83694887	C/T			Intron	
	3	**None Found**					
	4	chrC2:83703769	T / delT			Intron	
	4	chrC2:83703775	T/C			Intron	
	4	chrC2:83703876	T/C			Intron	
	4	chrC2:83704029	T/C			Intron	
	5	chrC2:83712162	G/A	No change		Exon	
	6	chrC2:83717084	C/T			Intron	
	6	chrC2:83717181	C/A			Intron	
	6	chrC2:83717180	C/insC			Intron	
	6	chrC2:83717181	A/C			Intron	
	6	chrC2:83717196	C/T			Intron	
	6	chrC2:83717228	G/A			Intron	
	7	chrC2:83721012	T/C			Intron	
	7	chrC2:83721018	C/T			Intron	
	7	chrC2:83721122	T/insATACCT			Intron	
	7	chrC2:83721181	A/G			Intron	
	8	chrC2:83721458	A/G			Intron	
	8	chrC2:83721889	C/A			Intron	
	9	chrC2:83726158	C/T			Intron	
	9	chrC2:83726168	C/T			Intron	
	9	chrC2:83726368	G/A			Intron	
	9	chrC2:83726564	A/G			Intron	

	10	**None Found**					
	11	chrC2:83731319	C/T			Intron	
	11	chrC2:83731366	T/C			Intron	
	12	**None Found**					
	13	**None Found**					
	14	**None Found**					
	15	**None Found**					
	16	chrC2:83740952	C/A			Intron	
	16	chrC2:83740954	C/T			Intron	
	17	**None Found**					
	18	chrC2:83747554	T/C			Intron	
	18	chrC2:83747903	G/T			Intron	
	19	chrC2:83757168	C/A			Intron	
	20	chrC2:83786172	G/A			Intron	
	21	**None Found**					
	22	chrC2:83828611	T/A			Intron	
	22	chrC2:83828855	T/C			Intron	
	23	**None Found**				Intron	
	24	**None Found**					
	25	chr2C:84020439	Del G			Intron	
	26	chr2C:84020441	T/A			Intron	
	27	chrC2:84023436	G/A			3’ UTR	
Traf2b	1	**None Found**					
	2	**None Found**					
	3	**None Found**					
	4	**None Found**					
	5	chrC2:83789071	T/A			Intron	
	5	chrC2:83789069	G (Deletion)			Intron	
	6	chrC2:83792653	A/G			Intron	
	7	**None Found**					
	8	**None Found**					

Supplementary Table 4. Testing Novel SNPs for Asociation with Type 2 Diabetes

Selected SNPs from Supp. Table 3 were tested in the diabetic and non-diabetic cats. Amplicon numbers, chromosome positions and base numbers are shown as in the legend of that Table. Genotypes for all SNPs were analysed using the "–assoc" function in Plink. A1 indicates the minor allele (i.e. least frequently observed based on the whole sample); F_A and F_U indicate the frequency of the minor allele in the affected and unaffected cats, respectively. The P value is shown calculated using Fisher's Exact test. The Odds Ratio (OR) was also calculated by Plink.

Gene	Amplicon	CHR	BP	A1	F_A	F_U	A2	CHISQ	P
ETV5	9	C2	83702092	C	0.40	0.25	T	0.45	0.50
ETV5	9	C2	83702188	C	0.40	0.13	T	1.68	0.20
ETV5	9	C2	83702263	T	0.32	0.35	C	0.14	0.71
ETV5	9	C2	83702303	A	0.40	0.13	G	1.68	0.20
LIPH	2	C2	84134822	C	0.10	0.67	A	5.61	0.02
LIPH	8	C2	84159595	T	0.50	0.40	C	0.18	0.67
LIPH	9	C2	84162155	T	0.21	0.03	C	18.65	1.57E-05
SLC8A1	2	C3	109937722	C	0.01	0.02	T	0.14	0.71
E2F6	3	C3	134630855	A	0.08	0.07	G	0.01	0.91
E2F6	4	C3	134632649	G	0.19	0.29	A	0.40	0.53
E2F6	6	C3	134636600	G	0.25	0.13	C	0.47	0.49
